# Evaluating the effect of aging on interference resolution with time-varying complex networks analysis

**DOI:** 10.3389/fnhum.2015.00255

**Published:** 2015-05-12

**Authors:** Pedro Ariza, Elena Solesio-Jofre, Johann H. Martínez, José A. Pineda-Pardo, Guiomar Niso, Fernando Maestú, Javier M. Buldú

**Affiliations:** ^1^Laboratory of Biological Networks, Centre for Biomedical Technology, Technical University of MadridMadrid, Spain; ^2^Department of Basic Psychology, Universidad Autónoma de MadridMadrid, Spain; ^3^Complex Systems Group, Technical University of MadridMadrid, Spain; ^4^Universidad del Rosario de ColombiaBogotá, Colombia; ^5^CINAC, HM Puerta del Sur, Hospitales de Madrid, Móstoles, and CEU-San Pablo UniversityMadrid, Spain; ^6^Laboratory of Neuroimaging, Centre for Biomedical TechnologyMadrid, Spain; ^7^Laboratory of Cognitive and Computational Neuroscience, Centre for Biomedical Technology, MadridSpain; ^8^Montreal Neurological Institute, McConnell Brain Imaging Centre, McGill UniversityMontreal, Canada; ^9^Biomedical Research Networking Center in Bioengineering Biomaterials and Nanomedicine (CIBER-BBN)Madrid, Spain; ^10^Complex Systems Group & GISC, Universidad Rey Juan CarlosMadrid, Spain

**Keywords:** complex networks of the brain, aging effects, magnetoencephalography (MEG), functional networks, synchronization

## Abstract

In this study we used graph theory analysis to investigate age-related reorganization of functional networks during the active maintenance of information that is interrupted by external interference. Additionally, we sought to investigate network differences before and after averaging network parameters between both maintenance and interference windows. We compared young and older adults by measuring their magnetoencephalographic recordings during an interference-based working memory task restricted to successful recognitions. Data analysis focused on the topology/temporal evolution of functional networks during both the maintenance and interference windows. We observed that: (a) Older adults require higher synchronization between cortical brain sites in order to achieve a successful recognition, (b) The main differences between age groups arise during the interference window, (c) Older adults show reduced ability to reorganize network topology when interference is introduced, and (d) Averaging network parameters leads to a loss of sensitivity to detect age differences.

## Introduction

Older adults show a decline in information-processing resources, such as working memory, and it is commonly accepted that aging adversely affects memory abilities. In this regard, age-related difficulties to suppress irrelevant information from distractors are evident during the active rehearsal of the to-be-remembered material (Hasher and Zacks, 1988; Madden et al., 2004; Gazzaley et al., 2008; Solesio et al., 2009; Solesio-Jofre et al., 2011, 2012; Salami et al., 2014). Compelling evidence from functional neuroimaging studies has suggested that the disproportionate susceptibility to interference in older individuals is accompanied by greater activity in prefrontal and parietal regions relative to their young counterparts. This over-recruitment is thought to be compensatory when it is accompanied by similar task performance across age groups (Grady et al., 1994; Cabeza et al., 1997). On the other hand, less activity in frontoparietal regions may also be shown in aging as task demands increase, which is associated with a drop in performance (Reuter-Lorenz and Cappell, 2008). Despite extensive evidence on focal brain activity, the human brain is primarily a network and complex cognitive functions, such as interference resolution, may be mediated by interactions among a set of functionally related areas rather than specific brain regions (McIntosh, 1999; Salami et al., 2014). Functional connectivity (Friston et al., 1993; Salami et al., 2014) refers to the interactions among spatially remote brain regions (Wang et al., 2010) and connections within specific networks involved in high level cognitive processes seem to be altered with age, which in turn might affect behavior (Grady, 2000; Andrews-Hanna et al., 2007; Damoiseaux et al., 2008; Grady et al., 2010; Clapp and Gazzaley, 2012; Salami et al., 2014). Specifically, a greater distractibility has been associated with reductions in connectivity between the prefrontal cortex and the parahippocampal area (Clapp et al., 2011), and within a more anterior network including the middle frontal gyrus, anterior cingulate, and basal ganglia (Salami et al., 2014). Additionally, Geerligs et al. (2014) compared connectivity changes between four different networks showing increased connectivity in older compared to young adults.

Altogether, current research suggests an age-related reorganization within and between specific networks. On the other hand, most of the studies have considered functional networks as static entities without investigating their evolution over time. Importantly, functional networks emerge, evolve and disappear according to the specific requirements of a given cognitive process and also in the absence of external stimulation (Hutchison et al., 2012). Only a few studies to date have focused on the temporal evolution of different neural networks (De Vico Fallani et al., 2008a,b; Dimitriadis et al., 2010; Bassett et al., 2011; Doron et al., 2012). In this regard, Valencia et al. (2008) showed stable small-word configurations (Watts and Strogatz, 1998) in combination with variations in functional connectivity at different time points and frequencies during a visual task. Network reconfigurations at larger time scales have been shown to be strongly correlated with learning (Bassett et al., 2011). Hence, the temporal evolution of simultaneous brain networks can help identifying the neural mechanisms promoting interference resolution with progressing age. In this regard, magnetoencephalography (MEG), is an ideal tool to explore the dynamical properties of whole-brain networks as it enables a direct measurement of brain magnetic fields from pyramidal neurons in the human cortex with optimal temporal resolution (i.e., milliseconds; Hämäläinen et al., 1993).

Two main principles might be considered when exploring the temporal dynamics of whole-brain networks, the so-called local specialization and global integration of functionally linked brain networks (Sporns et al., 2000; Bressler and Kelso, 2001; Friston, 2002; Wang et al., 2010; Zhu et al., 2012). In this context, graph theoretical approaches provide valuable tools to investigate the topological organization of large-scale functional networks supporting cognitive processes (Boccaletti et al., 2006; Bullmore and Sporns, 2009; Wang et al., 2010). Especially, they have been able to demonstrate that the healthy brain is organized according to a small-world architecture that favors cognitive performance (Smit et al., 2012), which is characterized by high local specialization (high clustering coefficient, C) and high global integration (short topological distance between nodes or path length, L; Watts and Strogatz, 1998). Additionally, deviant graph parameters have been used as markers for several pathological conditions (for a review, see Bassett and Bullmore, 2006; Guye et al., 2010; Wang et al., 2010; Stam and van Straaten, 2012), such as mild cognitive impairment and Alzheimer’s disease (Stam et al., 2007, 2009; de Haan et al., 2009; He et al., 2009; Buldú et al., 2011; Smit et al., 2012; Petti et al., 2013; Pineda-Pardo et al., 2014) and also for healthy aging (Micheloyannis et al., 2009). In this regard, healthy aging generally leads to alterations in the topology of large-scale functional networks with connectivity patterns more similar to “random” networks (Micheloyannis et al., 2009; Petti et al., 2013) and, hence, deviating from the optimal small-world organization observed in healthy young individuals.

Graph theory methods have been used to describe age effects on large-scale functional connectivity, predominantly at rest. Compelling evidence has reported age-related decreases in small-worldness of resting state networks in fronto-cingulo-parietal clusters (Achard and Bullmore, 2007; Meunier et al., 2009; Wang et al., 2010; Smit et al., 2012; Petti et al., 2013). However, only a few studies to date have examined age effects on task-related functional connectivity with graph approaches (Heitger et al., 2013), with only one of them focusing on memory processes (Wang et al., 2010). In this respect, the authors observed age-related disruptions of large-scale networks relevant to memory encoding and recognition. Specifically, older adults showed a widespread loss of long-range connections and longer path lengths in fronto-temporal and temporo-parietal regions with a few increases in posterior parietal regions.

The present study aimed at expanding limited previous work from graph approaches on age-related disruptions of memory-based functional connectivity, with special emphasis on the temporal evolution of network topology. To address this important issue, we examined whole-brain temporal dynamics of large-scale functional networks with MEG during the performance of an interference-based working memory task in young and older adults. To this end, we calculated phase synchronization (PS) across whole-brain regions and computed complex network parameters within each age group for alpha (8–12 Hz), beta (12–30 Hz) and gamma (30–48 Hz) bands, observing that the alpha band was the one reporting significant differences between young and older individuals. We then compared the abovementioned complex network values between age groups for successful recognitions. We also focused on the ability of functional networks to evolve and adapt in time during both the maintenance and interference period. Finally, we investigated network differences before and after averaging network parameters between both maintenance and interference windows. In contrast with previous research that focused on memory encoding and recognition, we were interested in memory maintenance (MM) as it corresponds to the period in which distraction is presented and interference resolution takes place. The present work is built on the study by Solesio-Jofre et al. (2011), who examined age-related changes in brain activations during MM. The authors demonstrated that interference resolution from distractors during the active maintenance of information requires greater neural resources for older adults in order to match the level of performance seen in young adults. Based on the initial analyses of Solesio-Jofre et al. (2011) and previous studies of network functionality in aging (Meunier et al., 2009; Madden et al., 2010), we hypothesized that older adults would demonstrate altered temporal dynamics in whole-brain functional networks during task performance compared to younger adults. To investigate this issue, we analyze how the topology of the functional networks of both young and old adults evolves during the experiment and we compare it with the classical analysis of the averaged functional networks, i.e., disregarding the fluctuations of the network topology along the experiment.

## Materials and Methods

### Participants

The sample comprised 20 healthy individuals divided into two groups according to their age, young and older adults (see **Table 1** for a description of the sample). They were selected from the adults program at the *Universidad Complutense de Madrid* (UCM). All participants reported corrected to normal vision and hearing within the normal range. All participants underwent a screening evaluation including a semi-structured interview, the reduced Geriatric Depression Scale rGDS (Yesavage et al., 1983), and the MiniMental State Examination MMSE (Folstein et al., 1975). They were required to satisfy a number of inclusionary criteria: (I) No psychiatric diagnosis described by the American Psychiatric Association (DSM-IV-TR axis I or II disorder), (II) No chronic neurological disease (e.g., seizure disorder or dementia) or severe medical illness that requires medication (e.g., diabetes or cardiopathies), (III) A score <5 on the rGDS, and (IV) A score >27 on the MMSE. Informed consent was obtained prior to participation and approved by the Institutional Review Board at UCM.

**Table 1:** Demography of the sample.

	Female/male	Age	MMSE	rGDS
Young	6/3	21.88 (3.40)	29.77 (0.44)	0.92 (1.38)
Older	9/2	64.45 (4.68)	29.17 (0.83)	1.58 (2.47)

Variables are included as mean values (standard deviation).

### Cognitive Task

**FIGURE 1:**
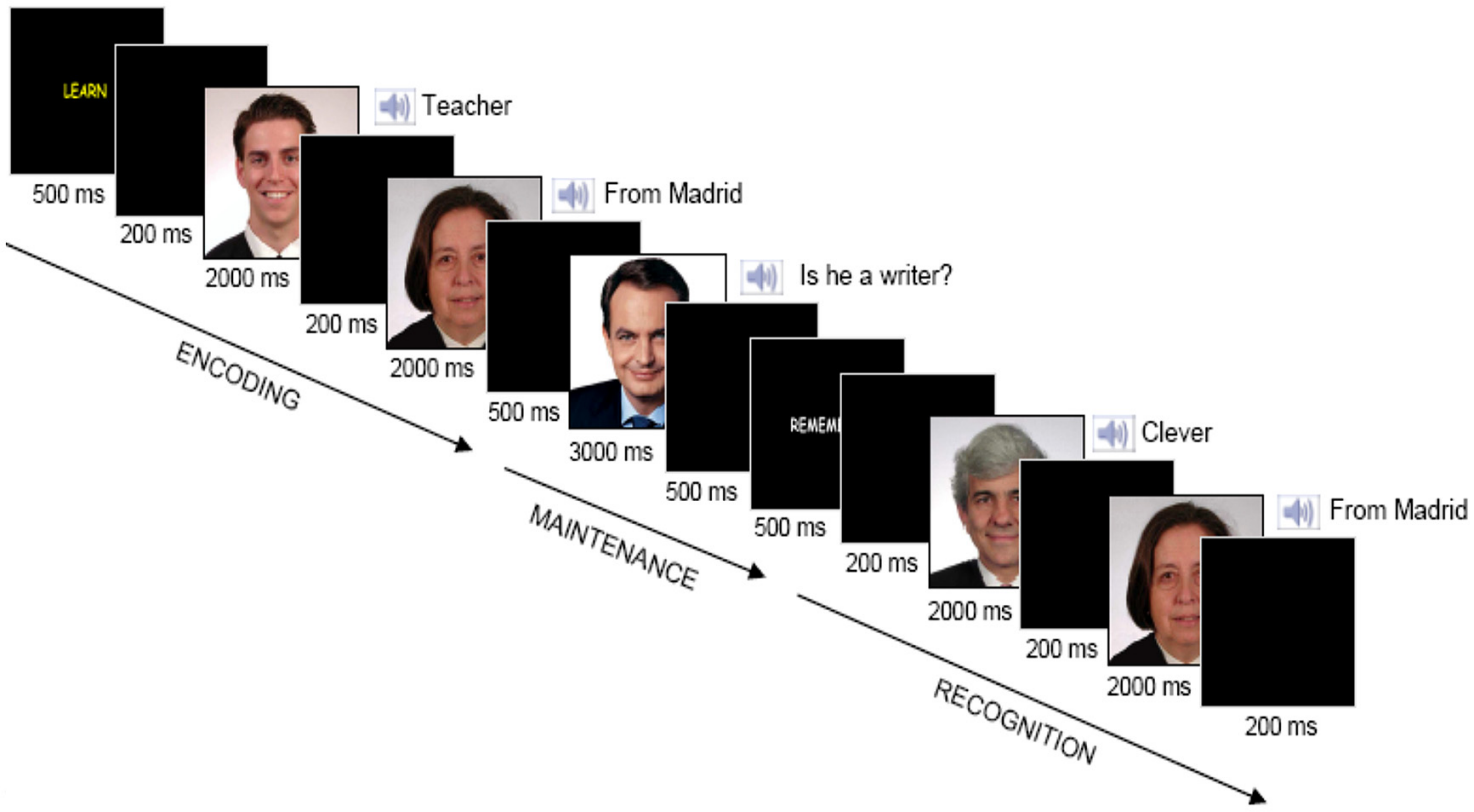
**Trial structure.** Two paired-associates were shown subsequently for 2000 ms each to memorize during the encoding stage. An interfering picture of a famous face was displayed for 3000 ms and subjects were asked about some attribute related to that picture during the maintenance stage. Two paired-associates were presented subsequently for 2000 ms each during the recognition stage and subjects reported whether each of them had appeared during the encoding phase.

We performed an interference-based working memory task, composed of 120 trials (see Solesio et al., 2009). Stimuli were presented using E-Prime 1.2 software (Groningen, The Netherlands). The experimental design is schematically depicted in **Figure 1**. It is divided into three stages: encoding, maintenance and recognition. A ‘LEARN’ yellow cue (500 ms) indicated the beginning of each trial, followed by a blank screen (200 ms). Two paired-associates, each of them composed of a visual stimulus (face) plus an auditory stimulus (semantic attribute describing some aspect of the face, i.e., ‘clever’), were subsequently shown during 2000 ms, separated by a blank screen (200 ms). Participants were instructed to memorize each pair. Next, after a 500 ms blank screen, an interfering face of a famous person was presented during 3000 ms. Participants had to answer a yes/no question (i.e., ‘Is he a writer?’) pressing one of two response buttons, followed by a blank screen (500 ms). Next, a ‘REMEMBER’ white cue appeared (500 ms), followed by another blank screen (200 ms). Thereafter, two more paired-associates were shown during 2000 ms each. Finally, another blank screen appeared for 200 ms. Subjects were required to make a match/non-match button-press response with the index finger to each probe as quickly as possible without sacrificing accuracy. We used a specially designed button panel and left/right (yes/no) index finger assignment was counterbalanced across participants. All participants were right-handed as stated with the initial semi-structured interview. On 120 of the 240 probes, the two paired-associates had been presented previously during the encoding period (a cue visual stimulus plus a cue auditory attribute) and the order of cue paired-associates at recognition was randomized. For the other 120 probes, the two paired-associates were foils. Specifically, 60 of these 120 probes were two paired-associates consisting of a cue visual stimulus plus a novel auditory attribute, and the other 60 consisted of a novel visual stimulus plus a cue auditory attribute. The presentation of ‘Old/New’ paired-associates was randomized and counter-balanced across all trials.

Visual stimuli consisted of colored pictures of human faces from the MEG laboratory database for faces. Sex and age were counter-balanced across all pictures. Age range was the same as the one employed in the selection of young and older participants (19–27 years for young participants, and 55–73 years for older participants). Famous faces were selected based on a preliminary behavioral study conducted with a different set of participants in which pictures were equally recognizable to young and older adults (Solesio-Jofre et al., under revision). Paired-associates and famous faces at the maintenance period were novel across trials. The set of employed images consisted of 240 faces for the encoding stage, 120 for the maintenance, and 240 for the recognition stage. From those at the recognition state, 120 were target (faces presented at encoding) and the rest were distractors. From the set of distractors, half incorporated a novel auditory stimulus and the half matched those at the encoding stage, but incorporated a novel face. Gender and age showed a variety of neutral expressions and were controlled across pictures.

Auditory stimuli were stereo-recorded at a frequency of 44.1 KHz and 16 bits. The set of stimuli consisted of 300 words: 100 were adjectives taken from the dictionary of the Royal Academy of the Spanish Language; 100 were professions taken from the Spanish National Institute of Statistics; and the 100 remaining were places of residency taken from the Spanish National Institute of Statistics. Prior to the MEG scan, subjects undertook a 20 trial-training session with the same structure as described above.

### Magnetoencephalografic Recordings

Magnetic fields were recorded using a 148-channel whole-head magnetometer (MAGNES® 2500 WH, 4-D Neuroimaging, San Diego, CA, USA) confined in a magnetically shielded room. The sampling rate was set to 678.17 Hz. An online anti-aliasing bandpass filter between 0.1 and 100 Hz was applied. Four electrodes were attached for the identification of blinks and eye movements; two of them near the left and right outer canthus and the other two above and below the right eye. Prior to the MEG measurements, the position of the magnetometers relative to the subject’s head was determined utilizing five small radiofrequency coils.

Responses at recognition were classified either as ‘hits’ (both answers were correct at recognition) or ‘errors’ (one answer or none of them were correct at recognition). We were interested in successful recognition; hence, we selected trials with hit responses for subsequent network analysis.

Baseline correction was applied on the basis of a pre-stimulus 100 ms window. Thereafter, the signal was submitted to a low-pass filter of 48 Hz. Ocular artifacts were corrected using BESA (version 5.1.6; MEGIS Software GmbH, Gräfelfing, Germany), which is a standard artifact-correction tool. Datasets were then visually inspected for movement artifacts, and epochs with peak-to-peak amplitudes exceeding a threshold of 3 pT were discarded from further analysis. In order to avoid any bias related to the different number of trials across subjects, we used a quality criterion referred to the minimum number of trials free of artifacts, which in the present study were 32. In individuals with more than 32 trials free of artifacts, we selected 32 of them randomly. From these trials, we segmented epochs of 1500 ms, including the blank-screen period prior to presentation of the interfering stimulus (500 ms) and the first 1000 ms after it. This segmentation was applied to avoid muscular artifacts coming from the button pressing while answering the interfering question. Hence, segmented epochs contained two different parts: (a) MM window (500 ms) and (b) and interference window (1000 ms).

### Synchronization Analysis

The construction of the functional networks relies on the evaluation of the PS between brain regions. PS detects when the phases of two signals synchronize, even though their amplitudes remain uncorrelated (Pikovsky et al., 2001; Pereda et al., 2005) and was quantified through the phase-locking value (PLV; Lachaux et al., 1999) using HERMES toolbox (Niso et al., 2013). Phases associated with the dynamics recorded at each magnetometer were extracted using the Hilbert Transform (Pikovsky et al., 2001). Next, we defined *φ(t)* as the difference between the two phases and calculate the PLV as:

(1) $$PLV=\;\langle e^{i\varphi \left(t\right)}\rangle =\sqrt{{\langle \cos\varphi (t)\rangle }^{2}+{\langle \sin\varphi (t)\rangle }^{2}}$$

The PLV index ranges from 0 to 1 and indicates how the relative phase is distributed over the unit circle. Higher PS between two signals is related to small differences between phases and high PLV, and vice versa for lower PS.

We were interested in the temporal evolution and topology of the functional networks. This way, we split the 1500 ms epochs into time windows of 50 ms length each with no overlap and evaluate the PLV within each temporal window. The window length was set to a value low enough to guarantee a sufficient number of points to observe a non-stationary temporal evolution of the network structure, but large enough to allow an accurate PLV. PLVs were computed in ten frequency bands (from 8 to 48 Hz, with central frequencies separated each 4 Hz) between pairs of the 148 magnetometers. Lower frequencies could not be considered due to edge effects after Hilbert transforms. Then, results were normalized with respect to a baseline (an open eyes resting state period of 100 ms). PLVs were then merged to form alpha (8–12 Hz), beta (12–30 Hz), and low gamma (30–48 Hz) frequency bands. Note that the length of the time windows to obtain the PLV is lower than the 1/f_min_ limit suggested by Leonardi and Van De Ville (2015). Nevertheless, the fact that we are analyzing non-stationary signals allow us to reduce the length of the time window beyond this limit, as recently explained by Zalesky and Breakspear (2015). Importantly, statistical differences between network parameters were only found at alpha band. Thus, we will focus on this frequency band in the forthcoming sections.

### Complex Network Analysis

The PLV between all pairs of channels led to a *NxN* (*N = 148*) symmetric matrix *W*, where its elements *w_ij_* quantify the PS between node *i* and node *j*. Note that the *W* matrix is the mathematical description of a weighted network, where the *N* nodes correspond to the brain regions whose activity has been recorded by the MEG and the weighted links account for how coordinated the activity between these brain sites is. Due to the time segmentation into 50 ms windows, we obtained a set of matrices *W* for each individual, and we tracked how the topology of these matrices changed with time. With this aim, we computed a series of complex networks parameters for each matrix of the *W* set: the network strength (*S*), outreach (*O*), weighted clustering coefficient (*C_w_*), global efficiency (*E_g_*), and average shortest path (*SP*; see Rubinov and Sporns, 2010, for a detailed description of all network metrics). **Table 1** summarizes the mathematical definition of these parameters, which were obtained, first, for each node of the network and, second, averaged over the whole network. This procedure was followed for each individual and, finally, each network metric was averaged over individuals of the same group, leading to an average of the ensemble with its corresponding error.

We have also computed the average values for the MM, the interference and the whole experiment, in order to evaluate the information gained from the analysis of the evolution of the network parameters.

### Statistical Analysis

Age-related differences in complex network parameters were calculated using non-parametric Kruskal–Wallis test (Siegel and Castellan, 1988). Reported *p*-values were corrected for multiple comparisons using a non-parametric permutation approach as elsewhere (Nichols and Holmes, 2002). Statistical significance was considered for *p*-values lower than 0.05.

## Results

### Functional Network Parameters in Alpha Band

**FIGURE 2:**
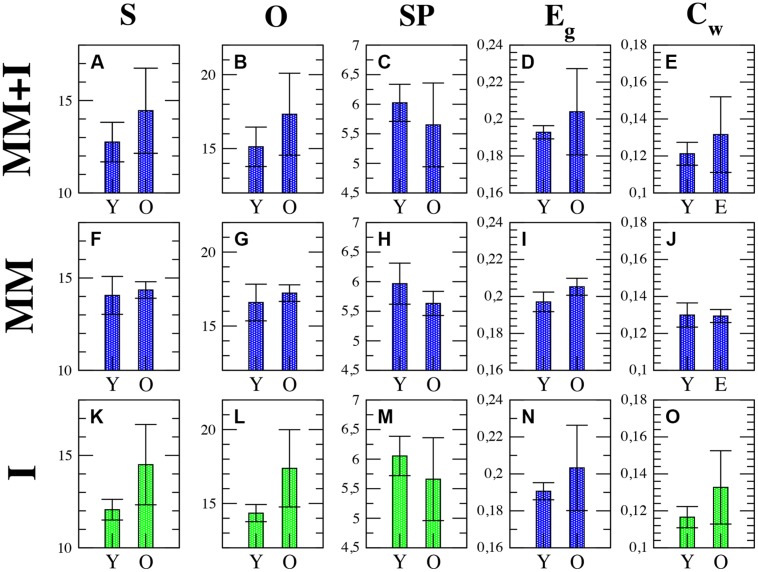
**Average network parameters obtained during the whole experiment MM+I (A–E), memory maintenance MM (F–J) and interference I (K–O) windows: strength (*S*), outreach (*O*), weighted clustering coefficient (*C_w_*), global efficiency (*E_g_*) and average shortest path (*SP*).** Rows refer, respectively, to MM+I, MM and I. Statistically significant parameters (*p* < 0.05) are highlighted in green, see **Table 3** summarizing the reported *p*-values. Black bars represent standard error of the mean. Y = young, O = old.

We have computed a group of classical network metrics over averaged and non-averaged functional networks in order to investigate functional activity (see **Table 2**). Our first approach was to obtain three different averages of the network parameters during: (A) MM, (B) interference (I), and (C) the whole experiment (MM+I). As we will see, the averaging of the network parameters leads in many cases to non-significant differences between groups, but we will use them as a reference to evaluate the advantages of analyzing their temporal evolution. First row of **Figure 2** shows the network parameters of the functional network obtained for averaging the set of synchronization matrices {W} during the whole experiment (MM+I). We observed that the older adults group had a higher average of the network strength S (**Figure 2A**), indicating a more synchronous activity during the whole experiment. As a consequence, the outreach *O*, measuring how the synchronized activity is correlated with the physical length of the links (see Materials and Methods) also had higher value in the older adults group (**Figure 2B**). Accordingly, the network shortest path *SP* was lower in the older group (**Figure 2C**), since the topological length of a link is obtained as the inverse of its weight (which, in turn, measures the synchronization). Thus, a higher value of S is translated into a lower average length of the network links, reducing the “topological distance” between nodes and leading to a lower *SP* in the older group. Note that this does not necessary indicate a better/worse organization of the network structure (in terms of information-processing) since the lower *SP* was just a consequence of having a larger *S* (i.e., higher average synchronization). The global efficiency E_g_ is a closely related measure of the overall connectivity of the network (Latora and Marchiori, 2001). *E_g_* was obtained as the inverse of the harmonic mean of the shortest distances between nodes, and it normally correlates with the inverse of *SP*, which is the case of our experiment (**Figure 2D**). As explained before, the higher efficiency of the older group is just a consequence of having a higher average synchronization between cortical regions.

**Table 2:** Mathematical definition of the network parameters: strength (S), outreach (O), weighted clustering coefficient (*C_w_*), global efficiency (*E_g_*), and average shortest path (*SP*).

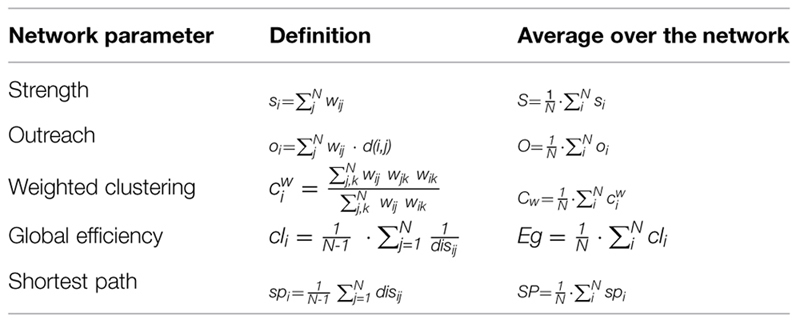

*w*_*ij*_* quantifies the synchronization between node *i* and node *j* computed with the PLV (see Materials and Methods), *d(i,j)* accounts from the physical distance between nodes *i* and *j*, and *dis_*ij*_* is the shortest topological distance to go from node *i* to node *J.* The node strength (*S*) accounts for the sum of the weights of the links arriving to node *i*. Outreach (*O*) is a network metric that combines the weight of the links of node *i* together with its length and it has been related with the energy demanded for maintaining functional connections (Buldú et al., 2011). The clustering parameter (C*_*w*_*) accounts for the functional triplets inside the network. The shortest path (*SP*) is a measure of the topological distance between all nodes of the networks and the global efficiency (*Eg*) averages the inverse of the shortest paths nodes.*

Finally, we focused on the local properties of the network by inspecting the weighted clustering coefficient *C_w_*, measuring how dense the connections are at the local level. In particular, how strong the connections forming triplets between brain regions are. We report a higher value in the older group (**Figure 2E**), which can be attributed again to a higher value in the average synchronization. Nevertheless, although there are clear differences at the average value, none of the network parameters had significant statistical differences between groups in the MM+I analysis (see **Table 3** for details), i.e., when an averaged functional network is considered for the whole experiment.

**Table 3:** *p*-values of the comparisons between average graph metrics after correction for multiple comparisons through non-parametric permutations test.

	S	O	SP	Eg	Cw
MM+I	0.0532	0.0524	0.0708	0.1008	0.0674
MM	0.8226	0.5496	0.3390	0.5026	0.8810
I	**0.0230**	**0.0174**	**0.0476**	0.704	**0.0264**

Values highlighted in bold are statistically significant (*p* < 0.05).

Second row of **Figure 2** shows the same network parameters but restricted to the MM window. We observe similar results as in MM+I, with the exception of the clustering coefficient where average values of both groups remain very close. The statistical analysis leaded to the same results as in MM+I: average differences between groups are not statistically significant.

Finally, in the third row of **Figure 2** the analysis is restricted to the interference window (I). In this case, *S* (**Figure 2K**), *O* (**Figure 2L**), *SP* (**Figure 2L**), and *C_w_* (**Figure 2O**) show statistically significant differences (*p* < 0.05) between young and older adults. The average strength *S* (*p* = 0.0230) during the interference period is higher for the older group, which suggests that they require a higher synchronization between cortical regions in order to successfully perform the memory task (note that only successfully recognized items are considered for the analysis). This fact is reflected in the differences in the SP: the increase of S reduces the topological distance between nodes, and SP now becomes statistically significant (*p* = 0.0476). Interestingly, the outreach is the network parameter showing the lower *p*-value (*p* = 0.0174) and, in turn, the largest difference between averages. When inspecting the local scale, we also obtain statistical significant differences in the clustering coefficient C (*p* = 0.0264). Only the *E_g_* does not show enough differences to overcome the statistical test, although the differences between the average values between groups are larger than in the MM window.

Two general conclusions can be extracted from the analysis of the topology of the averaged functional networks. First, a division of the task into MM and I gives more interesting information about the functional network structure as compared to the average over the whole experiment (MM+I). This is somehow expected as we are considering two different cognitive processes: MM and interference. Second, age differences are more evident during the interference period, leading us to consider that older individuals require higher synchronization between brain regions in order to perform a successful recognition. However, no correlations were observed between the averaged network metrics and the task behavior, i.e., the percentage of correct answers.

### Temporal Evolution of Functional Networks in Alpha Band

**FIGURE 3:**
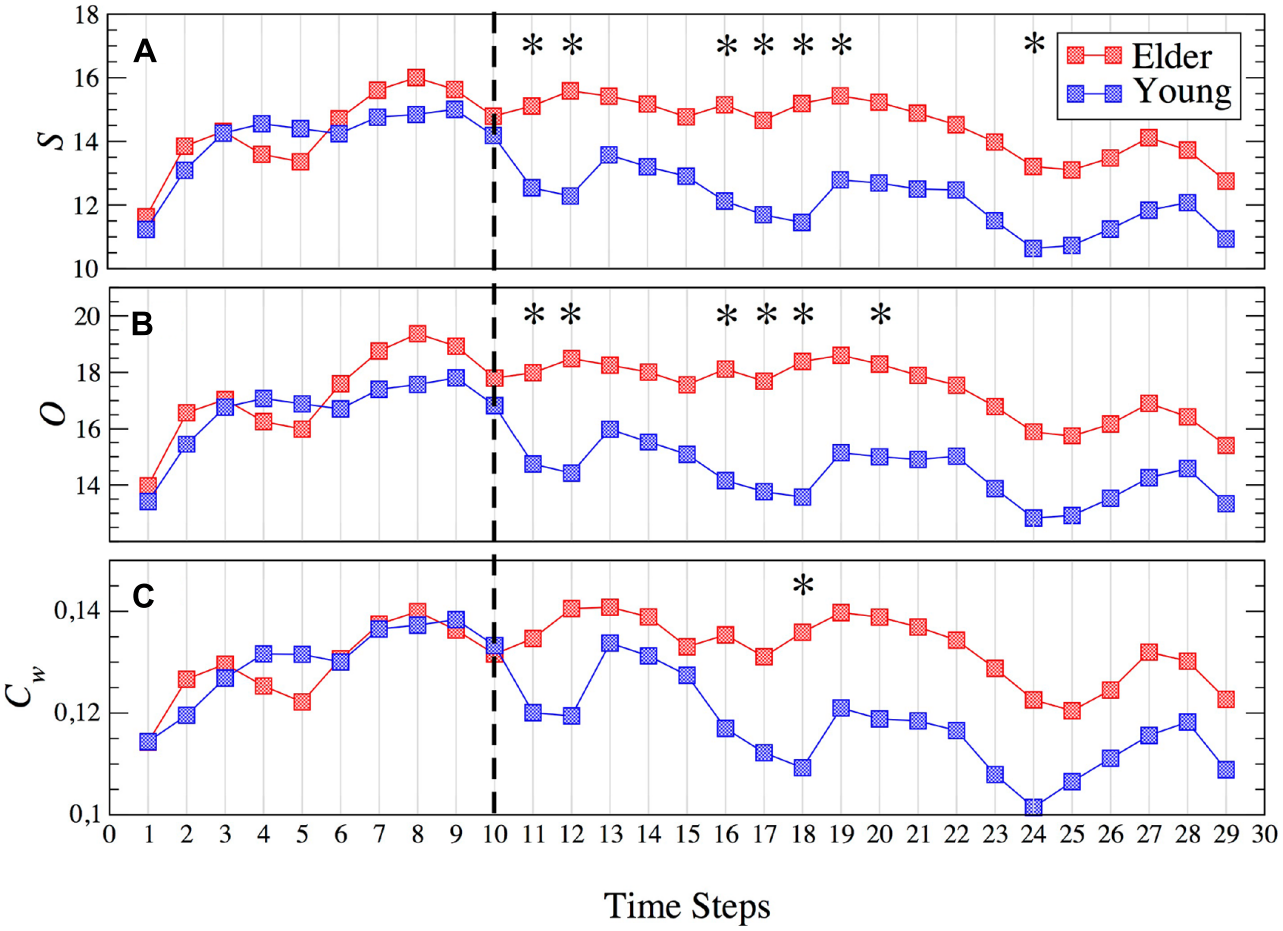
**Evolution of the network strength *S***(A)**, outreach *O***(B)** and weighted clustering *C_w_***(C)** for young (blue squares) and older (red squares) groups.** The dashed line indicates the end of the memory maintenance period and the beginning of the interference period. Stars indicate those time steps where the strength of the functional network shows statistically significant differences (*p* < 0.05) between both groups. See Supplementary Table S1 for a summary of the *p*-values of all parameters and time steps where statistically significant differences were found.

The fact that the topology of functional networks is not static recommends a study of how their temporal evolution is. Thus, it is desirable to split the whole experiment into short time intervals, calculate the properties of the functional networks at each interval and track their evolution. The shorter the time intervals, the larger the number of points and the better the temporal resolution. Nevertheless, the minimum length required to compute the synchronization between cortical regions (see Materials and Methods) introduces a lower threshold when dividing each time series into short windows. In our case, we have chosen a threshold of 50 ms, which leads to 30 points during the 1500 ms of each measurement. Next, we have obtained, and compared, all network parameters for each time step (see Supplementary Table S1 for the *p*-values associated to each network parameter and time step). **Figure 3A** shows the evolution of the network strength *S* during the whole experiment, with a dashed line indicating the end of the MM period and the beginning of the interference period. As we observed in the previous section, the average strength *S* was higher in older individuals. This phenomenon is clearly reported in the interference time-window, where *S_older_ > S_young_* over the whole window.

It is worth noting that those time steps where the functional network has statistically significant differences appear at the beginning of the interference period (see stars in **Figure 3A**, indicating the time steps with a *p*-value *p* < 0.05), vanishing after step 23 (700 ms after the beginning of the interference). Also note how the strength decreases during the interference period when compared with the MM period. This decrease is sharper at the beginning of the interference in the young group and much smoother in older individuals.

Similar results are obtained with the outreach *O* parameter, as shown in **Figure 3B**. Again the temporal evolution of the functional network reveals statistically significant differences between groups during the early stages of the interference period, in this case disappearing after 500 ms (step 20). For both groups, the outreach decreases in the interference period (see **Figures 2G,L**) following a similar profile as the network strength *S,* as it can be seen by comparing **Figure 3A** with **Figure 3B**.

Interestingly, the weighted clustering coefficient *C_w_* does not reveal such clear differences (see **Figure 3C**). The number of points that have statistically significant differences (stars in **Figure 3C**) decreases in just one time step located in the interference period. Again, the main differences are reported during interference and similar profiles to those of *S* and *O* are obtained, i.e., higher values during the MM in both groups and *C_w(older)_> C_w(young)_* during the interference.

**FIGURE 4:**
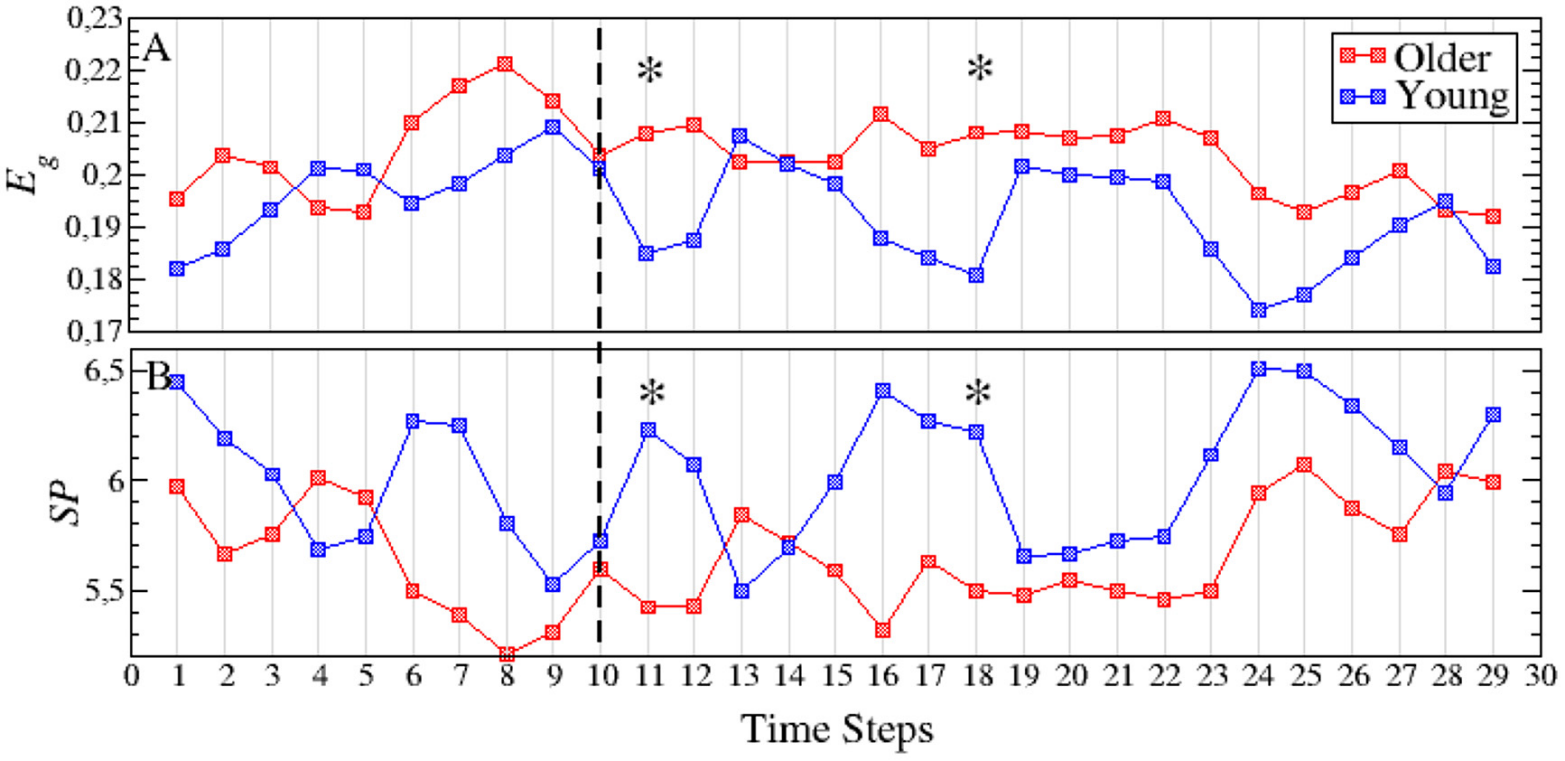
**Temporal evolution of the global efficiency *E_g_***(A)** and the average shortest path *SP***(B)** for both the young (blue squares) and older (red squares) groups.** The dashed line indicates the end of the memory maintenance period and the beginning of the interference period. Stars indicate those time steps where the strength parameter shows statistically significant differences between both age groups. See Supplementary Table S1 for a summary of the *p*-values of all parameters and time steps where statistically significant differences were found.

**Figures 4A,B** account for the two parameters related with the global transmission of information along the network: global efficiency *E_g_* and the average shortest path *SP*. As for the rest of parameters, differences between both groups are more evident in the interference period. In both measures, we only obtain two time steps with statistical differences, both of them during the first 500 ms of the interference region. The higher *E_g_* reported in the older group (**Figure 4A**) is a consequence of the higher value of *S*: the higher the synchrony between nodes, the higher the efficiency will be. Note that *E_g_* refers only to topological efficiency, and to the efficiency of the brain during the cognitive task. This decrease in network efficiency might indicate that older subjects require a higher synchronization between brain regions, which implies a higher energy demand, to achieve the same objective, in this case, a successful recognition after the inference.

Finally, we observe that SP behaves in the opposite direction from the topological efficiency parameter (**Figure 4B**). The increase of synchrony in the older adults group results in link weights of higher values that, in turn, lead to shorter paths between nodes (i.e., the higher the synchrony between two nodes, the shorter the distance between them).

### Fluctuations of Network Parameters in Alpha Band

**FIGURE 5:**
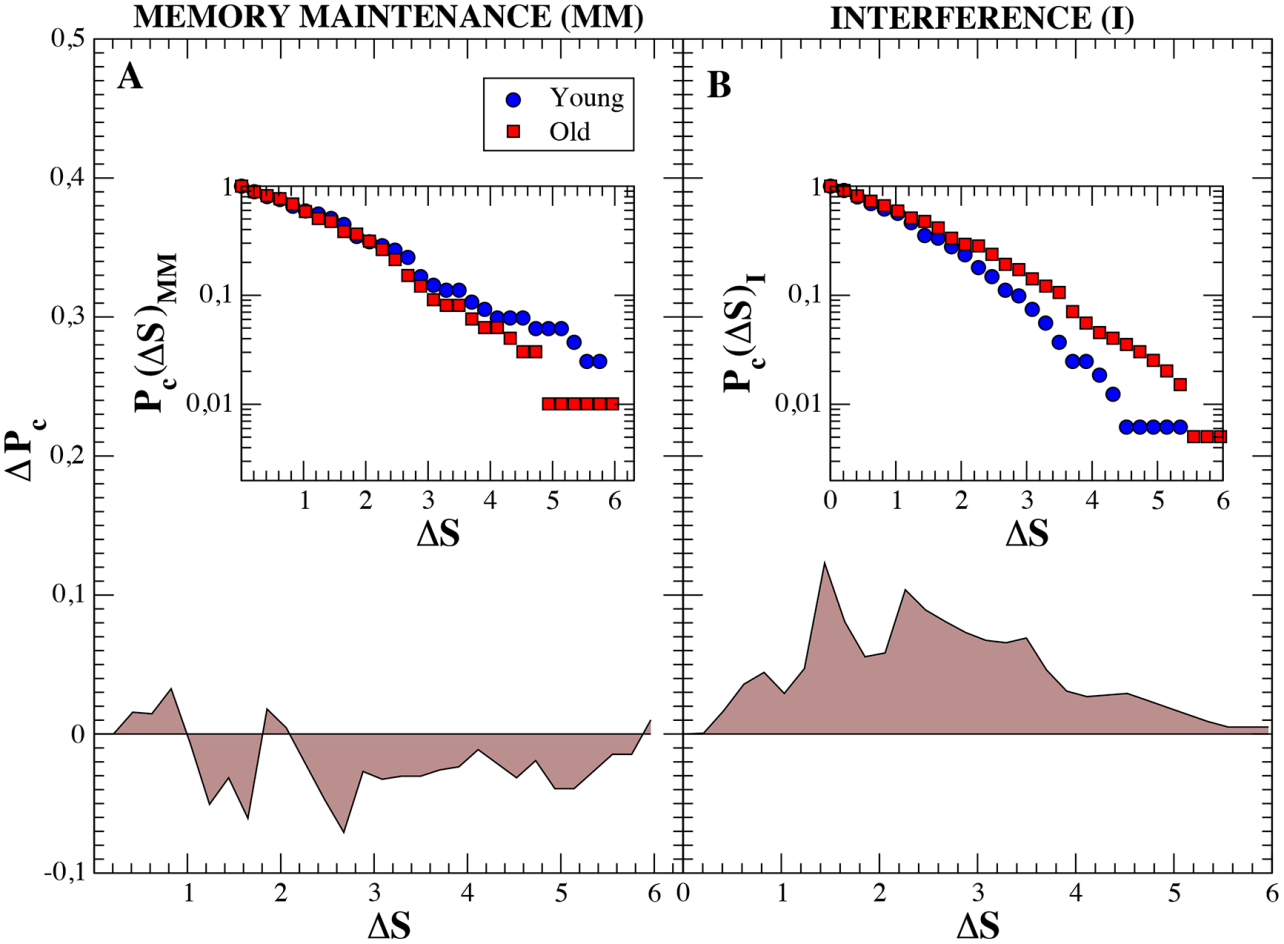
**Cumulative probability distributions of the increment of the network strength *ΔS* between consecutive time steps (insets) and differences between groups.** *ΔS* is defined as *ΔS = |S_i_-S_i-1_|.* Blue circles refer to *ΔS* of individuals belonging to the young group and red squares to *ΔS* of old subjects. Both insets show the cumulative probability distributions Pc(S) for the maintenance and interference regions (in log-linear scale), respectively. Main figures, **(A,B)** show the difference (P_c_^Old^ – P_c_^Y oung^), in linear scale, between both groups. We observe how, during the memory maintenance the increments of the network strengths are higher in the young group. On the contrary, during the interference region, fluctuations are mush larger in old individuals.

We finally focused on the ability of functional networks to evolve and adapt in time during both the MM and the interference window. With this aim, we quantified the increment of the network strength *S* at every time step *ΔS = | S_i_-S_i-1_|* since, as seen in the previous section, this parameter influences the rest. We used *ΔS* as an indicator of how much the synchrony over the functional network is able to increase/decrease during the short time interval of 50 ms associated to each time step. Next, we computed the cumulative probability distribution *P_c_(ΔS)* of having an increment of network strength higher than *ΔS*. *P_c_(ΔS)* was obtained for both groups separately. A comparison between MM and interference is plotted in **Figure 5**. Interestingly, during the MM, fluctuations of the network strength are higher in the young adults group (**Figure 5A**). When analyzing the same distributions during the interference period, we observe that the situation is reversed. In this case, the older adults group shows higher fluctuations of the network strength, which can be clearly observed by looking at the difference between both distributions (**Figure 5B**).

**FIGURE 6:**
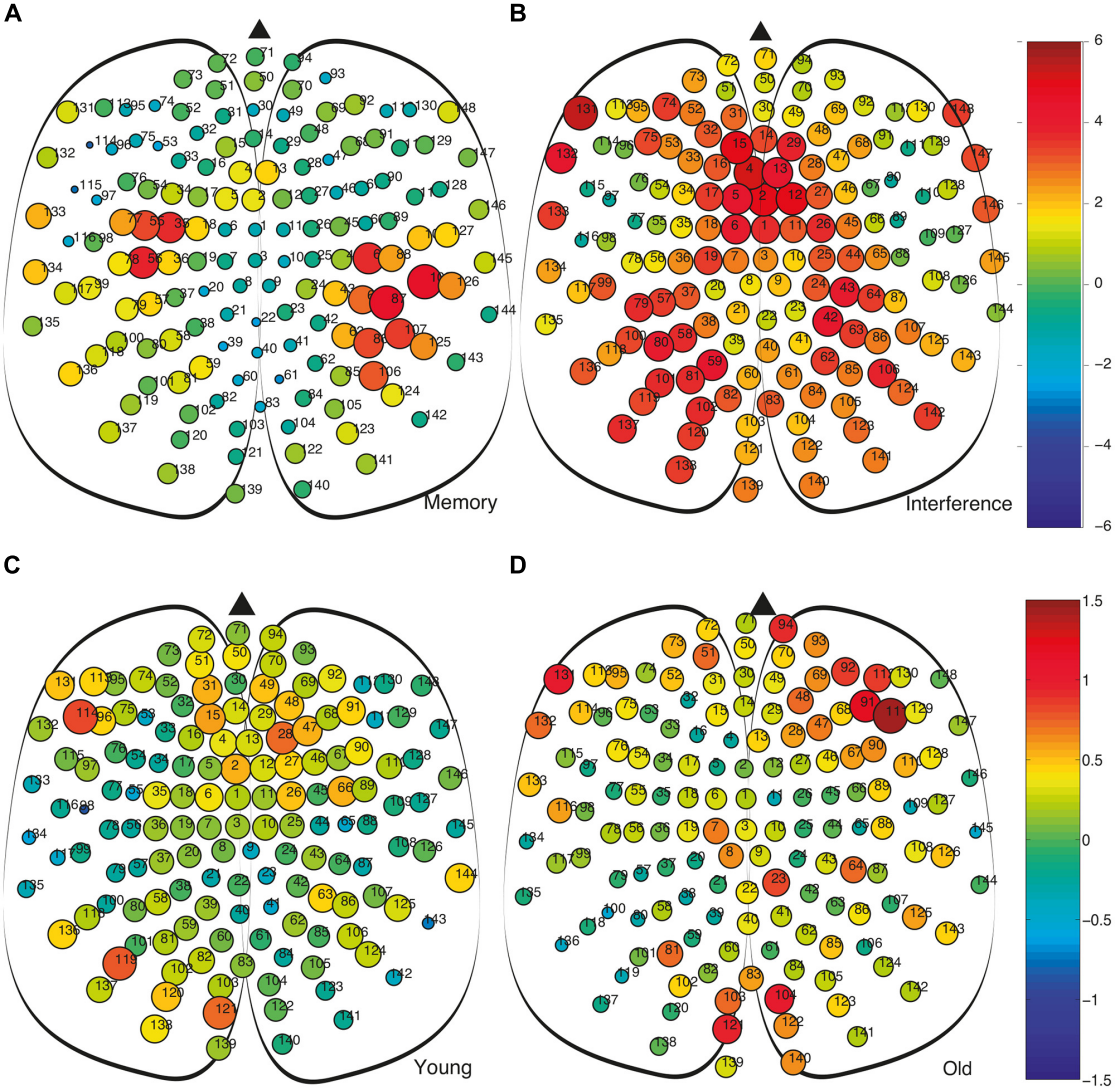
**Comparison of the strength of the nodes and their fluctuations during the memory maintenance and interference time windows. (A)** Comparison of nodal strengths s_i_ of both groups during memory maintenance (s_i_^old^ – s_i_^young^). **(B)** Same as differences as **(A)** obtained during the interference period. In **(A,B)** node size is proportional to the average node strength. We can observe how, during the interference period, the strength, in this case at the node level is much higher in the old group. **(C,D)** Average differences of strength between consecutive time steps *Δs = | s_i_-s_i-1_|* at the nodal level. Colors account for the difference of *Δs* between the memory maintenance and interference regions, i.e., *Δs^MM^ – Δs^I^*. This way, we compare the value of the fluctuations within the same group at the different regions (MM – I): **(C)** Young group and **(D)** old group.

We also analyse the fluctuations in strength at the nodal level. In **Figures 6A,B** we show between-group differences, in the average strength of the nodes (i.e., *Δs_old-young_ = s_i_^old^-s_i_^young^*), for both the MM and the interference time windows. During MM, we observe that despite the average network strength *S* is higher in the older adults group (see **Figure 2K**), this is not a generalized feature, since some nodes show a negative value of *Δs_old-young_* (**Figure 2A**). In the interference time-window, with a higher average network strength *S* for the older group compared to the young group (see **Figure 2K**), the majority of nodes have, accordingly, positive *Δs_old-young_*, with values much higher than the ones in the MM window.

Finally, it is worth analyzing strengths fluctuations at the node level during MM and interference. **Figures 6C,D** shows differences in strength fluctuations at the node level between both time windows (MM and I) for both age groups. This way, we first obtained strength fluctuations between two consecutive time steps *Δs = | s_i_-s_i-1_|* and, next, we averaged this value within each time-window and computed the difference *Δs^MM^ – Δs^I^*. The results show how in the older adults group, differences between MM and I are more extreme at certain node placed at the frontal and occipital regions (**Figure 6D**), while differences in the young adults group are more homogeneous (**Figure 6C**). In both cases, we have positive and negative deviations of *Δs^MM^ – Δs^I^*, indicating that it is worth going to the level of nodes to gain insights about how the fluctuations of the network strength are distributed among the cortical region.

## Discussion

We have investigated how functional networks of young and older individuals modulate their structure during an interference-based working memory task. We have seen that averaging the network topology during the whole experiment or, splitting it into two parts corresponding to the MM and interference, hide the differences reported between groups when a full segmentation along the experiment is performed. The latter analysis allows observing the evolution of the network parameters on time, reporting significant differences between young and older individuals at the beginning of the interference period. These differences are more pronounced in parameters such as the strength S or outreach O, despite they are also reported in the weighted clustering C_w_, average shortest path SP and network global efficiency E_g_. Interestingly, the ability of the network topology to reorganize is impaired in the older group, which shows lower variations of the network strength between consecutive time steps during the interference region when compared to the MM.

In the framework of functional brain networks, a diversity of studies have shown how network parameters such as small-worldness, synchronizability, robustness, or the existence of hierarchical community structures can be related to the processes occurring in the brain thanks to the interpretation coming from complex network theory (Bullmore and Sporns, 2009). At the same time, the effects of aging or the emergence of brain dysfunction can also be captured by the network metrics (Stam and van Straaten, 2012), as shown in different studies regarding normal aging (Micheloyannis et al., 2009; Zhu et al., 2012; Petti et al., 2013; Cao et al., 2014; Song et al., 2014), mild cognitive impairment (Buldú et al., 2011; Navas et al., 2014; Pineda-Pardo et al., 2014) or Alzheimer’s disease (Stam et al., 2007, 2009). Nevertheless, complex networks’ metrics have traditionally quantified the topological properties of networks with a unique and fixed value (Boccaletti et al., 2006). This approximation is valid in those cases where the time scales of the network evolution are orders of magnitude higher than the dynamical processes occurring in them. For example, when evaluating anatomical brain networks, it is reasonable to assume that the topology of the network is fixed during the measurement of the network itself, despite it is known that the synaptic connections also evolve with time (although at much slower rates). On the contrary, during a cognitive task, the brain activity suffers drastic changes at really fast time scales, and it is reasonable to expect that the associated functional networks could modify their topology during the task. Thus, averaging the network properties during a whole task may hinder information about the real topology of the functional network. Despite these limitations, the majority of the studies analyzing the topology of functional networks have dealt with, what we call, averaged functional networks (Bullmore and Sporns, 2009), which are obtained as the average of the activity during a cognitive process, e.g., one memory task, one single functional network. Unfortunately, this approximation may not give enough accuracy to draw conclusions about how the topology of the functional network is and how it evolves in unison with the cognitive task. In addition, it can be expected that the analysis of the evolution of a functional network, if possible, would result on a deeper knowledge of how the network arises, evolves and disappears, thus leading to a better understanding of the interplay between the network topology and cognitive processes.

Only a few studies have focused on the evolution of the topology of functional networks, most of them analyzing the effects of aging or adaptation during learning (Bassett et al., 2006; De Vico Fallani et al., 2008a,b). The fact that the majority of real networks change their topology as time goes by is capturing the attention on scientist working on complex networks analysis (Holme and Saramäki, 2012) and redefining classical measures in a way that they are able to capture the intrinsic evolution of the network structure.

In the current work, we use of this kind of methodologies in order to evaluate how young and older individuals perform an interference-based memory task and what are the consequences of undergoing an interference stimulus aiming to alter the MM. While the majority of the studies referring to functional brain networks considered them as static entities disregarding their temporal evolution, we focus specifically on this aspect, comparing the evolution of several network metrics during both the MM and interference. We show that calculating the parameters of the functional networks averaged along the whole experiment leads to differences between groups (young and older) that are not statistically significant. This fact reveals that, whenever it is possible, temporal averaging of the functional networks should be avoided. When the analysis is divided into two different stages, i.e., MM and interference, statistical difference between young and older individuals arise. Interestingly, it is only the interference window where the comparisons between network metrics show significant differences. This result indicates that at the whole-brain network level, the mechanism that allows to the elder group to achieve a successful recognition appears during the interference period, and this seems to be related with a global increase of functional connectivity, as observed for the network strength and outreach in the two first time windows. This result is in agreement with previous evidence considering the increase of functional connectivity as a compensation mechanism.

Interestingly, the fact that an impaired functional network shows a higher synchronization between its nodes has been reported in mild cognitive impairment (Buldú et al., 2011), a brain disease considered as Alzheimer’s prodromal state. The network metrics reflect differences between groups when taking into account both the global organization, by means of the outreach parameter, and the local organization, through the network clustering. The older group showed higher values of both metrics, indicating a higher activity of their functional networks, despite, as in the case of network strength, only the interference period shows significant statistical differences. The fact that outreach parameter takes into account the physical length of the links indicates that the differences between groups are also influenced by geometrical constraints. Nevertheless, we must note that the increase of the network strength in the older group has consequences in the rest of the network parameters. Higher strength leads to shorter distances between nodes, since topological distance scales with the inverse of the weight of the links. Thus, an increase of S is translated into a reduction (increase) in the SP (Eg). At the local scale, the clustering coefficient of the older group shows higher values than the young group, also capturing the enhancement of the synchronization in old individuals. As a general picture, the increase of the correlated activity of the older group modifies the network parameters accordingly, a fact that is mainly reported in the interference region.

In the present study, age-related differences in network topology are restricted to the alpha band. Converging evidence has demonstrated a central role of alpha oscillations in both the active processing necessary to MM (Palva et al., 2005; Sauseng et al., 2005) and functional inhibition (Klimesch et al., 2007; Jensen and Mazaheri, 2010). Both aspects are intrinsically linked to the notion of “top–down control,” which refers to an attentional control function that focuses on task-relevant information and suppresses task-irrelevant information by means of inhibition (Pinsk et al., 2004). In this regard, our results support the importance of alpha in inhibitory top–down control to enhance the retention of the to-be-remembered material and the suppression of interference during the maintenance period.

Finally, it is worth mentioning how the functional networks adapt their values in time. We observed how the increment of the network strength between consecutive time steps behaves differently during the MM and the interference periods. While the fluctuations of the network strength are slightly higher in the young adults group during the MM, this situation is reversed during the interference period, where fluctuations of the strength of the older adults group are much higher. This fact suggests that the ability of functional networks to maintain (modify) its topology during interference is decreased (increased) with aging, which may be related to inefficient top–down control, particularly, to deficits in inhibitory mechanisms necessary to override interference (Geerligs et al., 2012). These enhanced fluctuations of the network topology in order to compensate external disturbances might be of interest in the early diagnosis of neurodegenerative diseases such as Alzheimer’s disease or other types of dementia.

Finally, we should note that one limitation of our study is that the sample size is rather small, and thus, further work should be addressed to develop similar studies with larger populations. Nevertheless, the results presented here were statistically significant, and, in addition, are consistent with previous literature reflecting age-related changes during recognition processes (Grady et al., 1994; Cabeza et al., 1997).

## Conflict of Interest Statement

The authors declare that the research was conducted in the absence of any commercial or financial relationships that could be construed as a potential conflict of interest.
