# Supplementary material for: Evaluating the effect of aging on interference resolution with time-varying complex networks analysis
Source: Front Hum Neurosci. 2015 May 12;9:255. doi: 10.3389/fnhum.2015.00255 (PMC4428067; doi:10.3389/fnhum.2015.00255)
Supplement: Supplementary file 1 [file Presentation_1.PDF]

Supplementary Material

Evaluating the effect of ageing on interference resolution with time-varying complex networks analysis.

P. Ariza[a*], E. Solesio-Jofre[b*], J. Martínez-Huartos[c,d], J.A. Pineda-Pardo[e], G. Niso[f,g] F. Maestú[f] and J.M. Buldú[a,h]

a Laboratory of Biological Networks, Centre for Biomedical Technology, Madrid, Spain
b Department of Basic Psychology. Universidad Autónoma de Madrid, Madrid, Spain
c Complex Systems Group, Technical University of Madrid, Madrid, Spain
d Modelling and Simulation Laboratory, Universidad del Rosario de Colombia, Bogotá, Colombia
e Laboratory of Neuroimaging, Centre for Biomedical Technology, Madrid, Spain
f Laboratory of Cognitive and Computational Neuroscience, Centre for Biomedical Technology, Madrid, Spain
g McConnell Brain Imaging Center, Montreal Neurological Institute, McGill University, Montreal, Canada
h Complex Systems Group, Universidad Rey Juan Carlos, Madrid, Spain

* Authors P.A. and E.S-J. have contributed equally and must be considered as the first authors.

*** Correspondence:** Javier M. Buldú, Complex Systems Group, Universidad Rey Juan Carlos, Madrid, Spain
jmbuldu@gmail.com

1. Appendix 1: Definition of the Complex Networks Parameters

In our study, the mathematical representation of each functional is given by a symmetric matrix W

$$W = \begin{cases} w_{ij}, & if\ i\ links\ j \\ 0, & otherwise \end{cases}$$

The connectivity matrix W fully characterizes topological properties of a functional network with $N = 148$ nodes. The matrix is defined as weighted, which means that link weights range between zero and one ($0 \leq w_{ij} \leq 1$) and measure the Phase Locking Value (PLV) between each pair of electrodes. Based on the windowing segmentation (see main text), we obtain an ensemble $\{W\}$ of matrices per individual, whose topological changes will be captured by the analysis of their network properties. A detailed definition of the network parameters, already summarized in Tab. 2, follows:

Strength: As networks are weighted, s_i is the total sum of link's weights w_{ij} arriving to the node i^{th}. (Amaral et al., 2000; Newman, 2010).

Outhreach: It is defined as total sum of the product of link’s weights w_{ij} and their respective Euclidean or physical distances $d(i,j)$ (Buldú et al., 2011). In this case, $d(i,j)$ are the distances between nodes obtained

from the layout of the recording whole-head magnetometer (MAGNES® 2500 WH, 4-D Neuroimaging, San Diego, California, USA).

Weighted Clustering Coefficient: The triplets inside the networks c_i^w, represent the percentage of neighbours of a certain node that, in turn, are neighbours between them (Newman, 2010). In other words, it is a local parameter of the fraction of the triangles around an individual node. In case we want to obtain a global parameter, the average of local clustering coefficients lead to the C_w.

Averaged Shortest Path: Represents the average of the minimum number of nodes to be visited when going form node i to j (Newman, 2010). The minimum number of nodes, dis_{ij}, represents the minimum topological distance between nodes i and j. The average shortest path SP is obtained by applying the Disjkstra's algorithm to the distance matrix D, obtained as the inverse of the links' weights $\left(D_{ij} = \frac{1}{w_{ij}}\right)$.

Global Efficiency: It is defined as the harmonic mean of the inverse of the shortest path between all nodes of network (Latora et al., 2001). Global efficiency is related to the robustness of the network and to the integration of information due to its dependency on the shortest path. In other words, this concept refers to the ability of travel along the nodes of the network in the minimum number of steps. This measure, as well as the averaged shortest path, has been associated to functional integration of information on brain networks (Rubinov et al., 2010)

2. Supplementary Data

Time Window	S	O	SP	Eg	Cw
1	0.8248	0.6550	0.2600	0.3348	0.8792
2	0.4966	0.5508	0.2584	0.2604	0.6474
3	0.9396	0.7652	0.7006	0.6498	0.8288
4	0.8258	0.9426	0.7762	0.8910	0.6614
5	0.9380	0.8172	0.4618	0.4996	0.5496
6	0.4154	0.4148	0.2984	0.4178	0.8300
7	0.6602	0.6104	0.2600	0.4560	0.7168
8	0.4220	0.4272	0.0942	0.1746	0.7146
9	0.3778	0.3768	0.4220	0.6050	0.9410
10	0.4186	0.3440	0.7146	0.8864	0.6026
11	**0.0252**	**0.0080**	**0.0154**	**0.0342**	0.1850
12	**0.0342**	**0.0084**	0.0686	0.0546	0.1614
13	0.2632	0.3702	0.6614	0.5562	0.8768
14	0.1810	0.1818	0.8192	0.8766	0.3684
15	0.1198	0.1192	0.9404	0.8856	0.7616
16	**0.0152**	**0.0062**	0.0920	0.1138	0.1510
17	**0.0238**	**0.0184**	0.1114	0.1262	0.1062
18	**0.0016**	**0.0010**	**0.0344**	**0.0340**	**0.0040**
19	**0.0498**	0.0534	0.5604	0.5056	0.0946
20	0.0822	**0.0400**	0.7154	0.6082	0.1122
21	0.2310	0.2936	0.6076	0.6612	0.2992
22	0.2002	0.1970	0.3648	0.4084	0.2022
23	0.0834	0.1164	0.1132	0.1130	0.2264
24	**0.0362**	0.0778	0.0568	0.0532	0.0680
25	0.0768	0.0542	0.2230	0.2532	0.1468
26	0.0630	0.0808	0.2208	0.2566	0.1760
27	0.1610	0.1018	0.8870	0.7206	0.2656
28	0.5544	0.3082	0.4118	0.5356	0.8242
29	0.0982	0.1148	0.5122	0.3796	0.2318

Table S1. P values of the comparisons between graph metrics computed over time windows after correction for multiple comparisons through non-parametric permutations test. Memory maintenance goes from step 1 to step 10, while Interference goes from step 11 to step 29. P-values highlighted in bold are considered to be statistically significant ($p<0.05$).

Time Window	S	O	SP	Eg	Cw
1	11,2326	13,4123	6,4445	0,1821	0,1144
2	13,0902	15,4490	6,1910	0,1859	0,1196
3	14,2572	16,7686	6,0266	0,1934	0,1269
4	14,5533	17,0774	5,6825	0,2014	0,1316
5	14,4031	16,8871	5,7436	0,2010	0,1315
6	14,2389	16,7098	6,2690	0,1947	0,1300
7	14,7601	17,4000	6,2479	0,1982	0,1365
8	14,8437	17,5685	5,8061	0,2036	0,1373
9	15,0078	17,8081	5,5220	0,2090	0,1384
10	14,1853	16,8259	5,7275	0,2013	0,1333
11	**12,5421**	**14,7404**	**6,2288**	0,1849	0,1201
12	**12,2853**	**14,4161**	6,0693	0,1875	0,1194
13	13,5751	15,9916	5,4920	0,2072	0,1338
14	13,2038	15,5272	5,6965	0,2018	0,1312
15	12,9077	15,0790	5,9940	0,1982	0,1274
16	**12,1273**	**14,1563**	6,4094	0,1878	0,1170
17	**11,6942**	**13,7634**	6,2680	0,1843	0,1122
18	**11,4532**	**13,5687**	**6,2228**	**0,1811**	**0,1093**
19	**12,7912**	15,1413	5,6506	0,2017	0,1210
20	12,6925	**14,9951**	5,6642	0,1998	0,1189
21	12,5043	14,9017	5,7197	0,1996	0,1185
22	12,4752	15,0126	5,7446	0,1986	0,1166
23	11,5073	13,8697	6,1159	0,1858	0,1079
24	**10,6330**	12,8290	6,5083	0,1741	0,1015
25	10,7288	12,9237	6,4995	0,1771	0,1065
26	11,2459	13,5295	6,3347	0,1844	0,1111
27	11,8379	14,2553	6,1505	0,1904	0,1156
28	12,0785	14,5809	5,9443	0,1949	0,1182
29	10,9332	13,3353	6,3024	0,1824	0,1089

Table S2. Mean values of the network parameters for the group of young individuals. Those values with significant statistical differences are highlighted in bold. Memory maintenance goes from step 1 to step 10, while Interference goes from step 11 to step 29. Values highlighted in bold are considered to be statistically significant ($p<0.05$).

Time Window	σ(S)	σ(O)	σ(SP)	σ(Eg)	σ(Cw)
1	3,4174	4,0173	1,1346	0,0294	0,0338
2	3,9287	4,6014	1,0687	0,0278	0,0289
3	5,3425	6,3635	1,2512	0,0391	0,0374
4	4,6300	5,5573	0,8782	0,0301	0,0314
5	4,5201	5,4876	1,1471	0,0331	0,0326
6	5,4952	6,7075	2,0049	0,0496	0,0437
7	5,6057	6,7856	2,2102	0,0535	0,0465
8	3,7551	4,4081	1,6047	0,0371	0,0320
9	2,8494	3,2272	0,9944	0,0294	0,0256
10	2,4464	2,7713	1,0420	0,0269	0,0252
11	**2,1316**	**2,1595**	**1,0112**	0,0240	0,0235
12	**1,6268**	**1,5969**	0,5875	0,0160	0,0158
13	1,5411	1,7917	0,5458	0,0187	0,0149
14	2,1433	2,4222	0,9007	0,0263	0,0221
15	3,2066	3,5726	1,7001	0,0392	0,0344
16	**3,2792**	**3,7455**	2,1632	0,0388	0,0346
17	**2,4586**	**2,9133**	1,1710	0,0293	0,0258
18	**2,0748**	**2,5121**	**0,6022**	**0,0176**	**0,0186**
19	**2,9173**	3,3219	0,8406	0,0293	0,0249
20	2,6092	**2,8512**	0,7688	0,0225	0,0205
21	2,5822	3,0238	0,8768	0,0278	0,0224
22	2,8653	3,5331	0,8845	0,0292	0,0197
23	2,4960	3,0077	0,8875	0,0257	0,0196
24	**1,8476**	2,1885	0,7441	0,0202	0,0152
25	2,0105	2,2539	0,8660	0,0248	0,0145
26	2,7088	3,1608	1,1983	0,0331	0,0240
27	3,2449	4,0331	1,3027	0,0351	0,0273
28	3,1755	3,8503	1,1900	0,0322	0,0264
29	2,5340	3,1570	0,9864	0,0252	0,0215

Table S3. Standard deviation σ of the network parameters for the group of young individuals. Memory maintenance goes from step 1 to step 10, while Interference goes from step 11 to step 29. Values highlighted in bold are considered to be statistically significant ($p<0.05$).

Time Window	S	O	SP	Eg	Cw
1	11,6310	13,9653	5,9710	0,1952	0,1143
2	13,8481	16,5689	5,6647	0,2036	0,1267
3	14,3121	17,0413	5,7556	0,2014	0,1296
4	13,5944	16,2643	6,0125	0,1937	0,1253
5	13,3597	15,9933	5,9208	0,1928	0,1222
6	14,7074	17,5967	5,4978	0,2099	0,1307
7	15,6091	18,7521	5,3887	0,2170	0,1375
8	16,0069	19,3676	5,2072	0,2209	0,1399
9	15,6320	18,9230	5,3087	0,2142	0,1363
10	14,7918	17,7979	5,5983	0,2037	0,1316
11	**15,1097**	**17,9851**	**5,4209**	0,2080	0,1347
12	**15,5913**	**18,4864**	5,4252	0,2095	0,1405
13	15,4216	18,2521	5,8408	0,2024	0,1408
14	15,1733	18,0111	5,7094	0,2023	0,1389
15	14,7693	17,5603	5,5896	0,2026	0,1330
16	**15,1486**	**18,1151**	5,3160	0,2114	0,1354
17	**14,6607**	**17,6854**	5,6295	0,2048	0,1311
18	**15,1908**	**18,3872**	**5,4934**	**0,2080**	**0,1359**
19	**15,4333**	18,6036	5,4750	0,2084	0,1397
20	15,2314	**18,2894**	5,5423	0,2068	0,1389
21	14,8893	17,8918	5,4922	0,2075	0,1369
22	14,5204	17,5428	5,4548	0,2107	0,1343
23	13,9781	16,7909	5,4909	0,2068	0,1288
24	**13,2125**	15,8937	5,9449	0,1964	0,1226
25	13,1031	15,7468	6,0685	0,1929	0,1205
26	13,4780	16,1747	5,8750	0,1967	0,1245
27	14,1186	16,9025	5,7492	0,2008	0,1320
28	13,7286	16,4291	6,0373	0,1935	0,1302
29	12,7516	15,4031	5,9961	0,1920	0,1227

Table S4. Mean values of the network parameters for the group of older individuals. Those values with significant statistical differences are highlighted in bold. Memory maintenance goes from step 1 to step 10, while Interference goes from step 11 to step 29. Values highlighted in bold are considered to be statistically significant ($p<0.05$).

Time Window	σ(S)	σ(O)	σ(SP)	σ(Eg)	σ(Cw)
1	2,9352	3,4189	1,2626	0,0302	0,0308
2	3,8687	4,5194	1,1021	0,0341	0,0342
3	3,9925	4,7114	1,1703	0,0351	0,0330
4	3,6457	4,3867	1,2250	0,0364	0,0286
5	2,5479	3,0722	0,9323	0,0252	0,0200
6	3,1615	3,8530	1,3323	0,0342	0,0269
7	3,6550	4,4679	1,6099	0,0396	0,0330
8	3,4278	4,2117	1,1427	0,0373	0,0311
9	3,0060	3,6446	0,9485	0,0304	0,0272
10	3,0790	3,7773	0,8986	0,0305	0,0278
11	**2,2912**	**2,7330**	**0,5092**	0,0185	0,0231
12	**3,2024**	**3,7389**	0,5813	0,0228	0,0295
13	4,4578	5,1091	1,3987	0,0401	0,0400
14	3,5231	4,0533	0,9845	0,0321	0,0279
15	2,8564	3,2103	0,6716	0,0243	0,0235
16	**2,3828**	**2,7081**	0,5115	0,0189	0,0187
17	**2,9402**	**3,5235**	1,1502	0,0351	0,0235
18	**2,4317**	**2,9110**	**0,9336**	**0,0310**	**0,0189**
19	**2,7984**	3,4684	0,7966	0,0261	0,0215
20	3,4131	**4,1002**	0,8567	0,0309	0,0288
21	3,4177	4,2820	0,7130	0,0268	0,0295
22	3,0358	3,9876	0,9348	0,0304	0,0300
23	2,6165	3,1046	0,6380	0,0242	0,0261
24	**3,0411**	3,7811	1,3160	0,0341	0,0263
25	3,1075	3,8977	1,3129	0,0356	0,0242
26	2,1130	2,6957	1,0396	0,0249	0,0167
27	2,3143	2,4381	0,7804	0,0228	0,0222
28	3,3796	3,6155	0,9525	0,0321	0,0331
29	1,8229	2,1233	0,7557	0,0204	0,0146

Table S5. Standard deviation σ of the network parameters for the group of older individuals. Memory maintenance goes from step 1 to step 10, while Interference goes from step 11 to step 29. Values highlighted in bold are considered to be statistically significant (p<0.05).

References

Buldú. JM, Bajo. R, Maestú. F, Castellanos. N, Leyva. I, Gil. P, et al. (2011) Reorganization Of functional networks in mild cognitive impairment. PLoSOne6:e19584.

Rubinov M, Sporns O (2010) Complex Network measures of brain connectivity: uses and interpretations.Neuroimage.52(3):1059-°©-69.

Amaral LAN, Scala A, Barthélemy M, Stanley HE (2000) Classes of small-world networks. *Proc Natl Acad Sci USA* **97**:11149–11152.

Latora V, Marchiori M (2001) Efficient behavior of small-world networks. *Phys Rev Lett* **87**:198701.

Newman MEJ (2010) *Networks: an introduction*. Oxford University Press, New York.
